# Valuation of scleroderma and psoriatic arthritis health states by the general public

**DOI:** 10.1186/1477-7525-8-112

**Published:** 2010-10-01

**Authors:** Dinesh Khanna, Tracy Frech, Puja P Khanna, Robert M Kaplan, Mark H Eckman, Ron D Hays, Shaari S Ginsburg, Anthony C Leonard, Joel Tsevat

**Affiliations:** 1 Division of Rheumatology, Department of Medicine, David Geffen School of Medicine, University of California at Los Angeles, Los Angeles, California, USA; 2 Department of Health Services, School of Public Health, David Geffen School of Medicine, University of California at Los Angeles, Los Angeles, California, USA; 3 Division of Rheumatology, Department of Medicine, University of Utah, USA; 4 Division of General Internal Medicine, Department of Internal Medicine, University of Cincinnati College of Medicine, Cincinnati, Ohio, USA; 5 Center for Clinical Effectiveness, University of Cincinnati, Cincinnati, Ohio, USA; 6 Division of General Internal Medicine, Department of Medicine, David Geffen School of Medicine, University of California at Los Angeles, Los Angeles, California, USA; 7 RAND, Santa Monica, California, USA; 8 Veterans Affairs Medical Center, Cincinnati, Ohio, USA

## Abstract

**Objective:**

Psoriatic arthritis (PsA) and scleroderma (SSc) are chronic rheumatic disorders with detrimental effects on health-related quality of life. Our objective was to assess health values (utilities) from the general public for health states common to people with PsA and SSc for economic evaluations.

**Methods:**

Adult subjects from the general population in a Midwestern city (N = 218) completed the SF-12 Health Survey and computer-assisted 0-100 rating scale (RS), time trade-off (TTO, range: 0.0-1.0) and standard gamble (SG, range: 0.0-1.0) utility assessments for several hypothetical PsA and SSc health states.

**Results:**

Subjects included 135 (62%) females, 143 (66%) Caucasians, and 62 (28%) African-Americans. The mean (SD) scores for the SF-12 Physical Component Summary scale were 52.9 (8.3) and for the SF-12 Mental Component Summary scale were 49.0 (9.1), close to population norms. The mean RS, TTO, and SG scores for PsA health states varied with severity, ranging from 20.2 to 63.7 (14.4-20.3) for the RS 0.29 to 0.78 (0.24-0.31) for the TTO, and 0.48 to 0.82 (0.24-0.34) for the SG. The mean RS, TTO, and SG scores for SSc health states were 25.3-69.7 (15.2-16.3) for the RS, 0.36-0.80 (0.25-0.31) for the TTO, and 0.50-0.81 (0.26-0.32) for the SG, depending on disease severity.

**Conclusion:**

Health utilities for PsA and SSc health states as assessed from the general public reflect the severity of the diseases. These descriptive findings could have implications regarding comparative effectiveness research for tests and treatments for PsA and SSc.

## Introduction

Skin and joint disorders can substantially impact physical and psychological function. Psoriatic arthritis (PsA) and scleroderma (SSc) are 2 such disorders having varying degrees of severity and functional impairment, potentially resulting in long-term work disability [1,2]. Although incidence and prevalence rates vary in the literature, PsA is thought to affect 200-1000 per million people, and SSc 300-700 people per million [3,4]. PsA is characterized by a specific pattern of inflammatory joint disease, negative rheumatoid factor serology, and hyperkeratotic plaques that usually occur on the elbows, knees, and scalp. People with PsA often have extra-articular inflammatory features involving nail beds, entheses, and the uveal tract. Although there is no relationship between the degree of skin involvement and the severity of psoriatic arthritis, both aspects of this disease have important implications for its sufferers [5]. Similarly, people with SSc have varying degrees of both skin hardening and systemic involvement, which may include arthritis. SSc is categorized as limited SSc or diffuse SSc, depending on the extent of skin involvement [6]. Patients with limited SSc generally have a more favorable outcome, with a 5-year survival rate as high as 86% [7]. Diffuse SSc is characterized by rapid skin thickening and potentially severe pulmonary, cardiac, renal, and gastrointestinal involvement occurring in the first 3-5 years of the disease [6].

Despite disparate prognoses due to their systemic effects, both PsA and SSc have a detrimental effect on patient's health-related quality of life (HRQoL) [8,9]. There are 2 standard approaches to assessing HRQoL: 1) the health status approach, which describes functioning and the impact of illness on specific domains of health (e.g., physical functioning and pain, as captured by measures such as the SF-12 Health Survey), and 2) the value/preference/utility approach, which assesses the value or desirability of health states by having participants take hypothetical risks or make hypothetical tradeoffs among health states and summarizes HRQoL in a single number [10-12]. Health value measures include the time tradeoff (TTO) and standard gamble (SG). The TTO ascertains one's willingness to sacrifice longevity for better health. The SG ascertains one's willingness to undergo risky treatments in order to improve health. Health values can be assessed either directly from subjects or indirectly through health state classification systems, which map community-derived utilities onto subjects' health states. Health utilities most often serve as quality-of-life weights for calculating quality-adjusted life years (QALYs) in decision and cost-effectiveness analyses [13].

Utilities have been assessed directly both from patients with psoriasis and SSc [2,14]. The U.S. Public Health Service Panel on Cost-Effectiveness in Health and Medicine, however, has recommended using utilities derived from the general public, rather than from patients, for cost-effectiveness analyses [15]. Our objective was to assess utilities from the general public for health states common to people with PsA and SSc in order to provide "off-the-shelf" community quality-of-life weights for future decision and cost-effectiveness analyses involving diagnostic strategies or treatments for PsA and SSc. By excluding patients with these conditions, the utility of these health states from a societal standpoint could be assessed.

## Methods

### Study Subjects

We recruited 218 subjects age 18 years or older from Cincinnati, Ohio through flyers, posters posted at the University of Cincinnati and local grocery stores, and advertisements in local newspapers. Because patients with inflammatory arthritides suffer symptoms of joint pain and swelling and have difficulty in carrying out avocational activities and activities of daily living, and because the purpose of the project was to assess health values from the societal perspective (that is, from people by-and-large not familiar with the health states under study), we chose not to include people who had inflammatory arthritis. Thus, all subjects who did not have a history of inflammatory arthritides such as PsA, SSc, or rheumatoid arthritis (patients with osteoarthritis and fibromyalgia were allowed to participate), and who were able to read English were eligible. The protocol was approved by the University of Cincinnati Institutional Review Board and all subjects provided informed consent. Subjects received $30 gift cards for participating.

### Questionnaires

All subjects completed the questionnaires in face-to-face structured interview. Subjects first answered demographic questions about their age, sex, ethnicity, marital status, household income, and highest level of education attained. Participants' health status was assessed by using the SF-12 [16-18], a generic health status measure consisting of 12 items assessing 8 domains or subscales [16]. The 8 SF-12 subscales can be summarized into a Physical Component Summary (PCS) and a Mental Component Summary (MCS) score. Summary scores are normed to the U.S. general population, where the mean score is 50 and the standard deviation is 10. We used version 2 of the SF-12 and a standard (4-week) recall period.

#### Description of Health States

Each subject was given a brief description of PsA and SSc health states (Appendix) and asked to imagine how it would be to spend the rest of their life in that health state. We developed a total of 3 PsA and 5 SSc health states by using health state attributes from the Quality of Well-Being Self-Administered (QWB-SA) scale, a health state classification measure [19], supplemented by our own descriptions of skin and lung disease. The QWB-SA includes an exhaustive set of health outcome states and has been used in a variety of studies. Normative data on the QWB-SA are available [20-22]. The 3 PsA health states were: mild PsA, moderate PsA, and severe PsA. Severity was categorized by ability to perform major activities and self-care activities; the degree of skin involvement with psoriasis was not varied among PsA states. The 5 SSc health states were: mild SSc, moderate SSc, moderate SSc with lung disease, severe SSc, and severe SSc with lung disease. We specifically included lung disease in 2 of the SSc health states because lung disease (due either to pulmonary hypertension, interstitial lung disease, or both) is the leading cause of death in patients with SSc and because new therapies for lung disease have been approved recently or are being studied in clinical trials. We chose not to differentiate between pulmonary hypertension and interstitial lung disease for the SSc health states, as breathlessness is a common symptom for both conditions. Each subject rated 3 of the 5 randomly selected hypothetical SSc states, grouped according to the type of SSc (limited versus diffuse). In other words, participants valued 3 limited SSc disease health states or 3 diffuse SSc disease health states.

#### Utility Measures

Health utilities were elicited by a trained interviewer (S.G.) using U-Maker, a computer-assisted utility assessment software package [23]. Details of the assessment procedure have been published previously [2]. Briefly, subjects first rated the health states on a health rating scale (RS), which was presented as a "feeling thermometer" with scores ranging from 0 (dead) to 100 (perfect health). Next, participants completed a TTO exercise, which was represented graphically as a choice between 2 horizontal bars, 1 representing the full life expectancy in a given PsA or SSc health state (followed by death) and the other representing a given number of years (less than or equal to the life expectancy) in perfect health followed by death [23]. Based on the age of the subject, U-Maker utilized the life expectancy reported in U.S. life tables, rounding the life expectancy to the nearest 5 years [24]. The number of years in perfect health vs. in the PsA or SSc health state was varied in a "bisection" fashion until the patient no longer had a clear preference between living in the given health state or living the given amount of time in perfect health [25]. The TTO score was calculated by dividing the number of years of perfect health at the indifference point by the full life expectancy.

The final utility task was the SG. Participants were shown two circles: one was labeled with the PsA or SSc health state in question and remained the same on all of the screens; the second circle represented "perfect heath." The subject was offered a choice between living the remainder of his/her life in the given PsA or SSc health state vs. taking a gamble in which the 2 outcomes were perfect health for the remainder of life or immediate death [26]. Initially, the second circle was displayed as a pie chart with a 100% probability of perfect health. Assuming the subject preferred perfect heath in that scenario, the probabilities of perfect health and death in the second circle were then varied systematically by using bisection until the patient was indifferent between the certainty of life in the PsA or SSc health state or the gamble. The SG score was calculated by the following formula: 1 - the maximum acceptable probability of death.

#### Comprehension and Empathy

At the end of the health utility exercise, we asked the subjects to rate the clarity of the computer program on a 5-point response scale: "very confusing," "confusing," "neither confusing nor clear," "clear," or "very clear." In addition, subjects were asked if they were able to imagine themselves as the person in the hypothetical health states according to a 3-point scale: "very much," "a little bit," or "not at all."

### Statistical Analysis

Descriptive statistics for continuous variables are presented as means and standard deviations. We assessed normality of health utility measures by using the Shapiro-Wilk test; the RS scores for PsA and SSc health states were normally distributed but the TTO and SG scores were not. Nevertheless, because mean values are used in calculating QALYs, we present the data as means (SDs) in the text and the tables. Categorical variables are presented as frequencies and proportions in a contingency table format. Unadjusted comparisons for categorical outcomes were made by using chi-square and Fisher's Exact tests.

Because there were no statistically significant differences in utilities for limited vs. diffuse disease within the mild SSc, moderate SSc, severe SSc, moderate SSc with lung disease, and severe SSc with lung disease states (P-values ranged from 0.08 for RS scores for the severe limited vs. severe diffuse SSc subtype to 0.90 for SG scores for severe limited vs. severe diffuse SSc with lung disease), we merged results for limited with diffuse by each SSc severity category, e.g., moderate limited SSc with moderate diffuse SSc. All analyses were performed by using STATA software, version 9.2 (College Station, Tex.); P < 0.05 was considered indicative of statistical significance.

## Results

### Subjects' Characteristics

The mean (SD) age of the participants was 46.0 (12.9) years; 135 (62%) were female, 143 (66%) were Caucasian, and 62 (28%) were African-American (Table 1). Almost all subjects 212 (98%) graduated from high school and 155 (71%) had household incomes exceeding $25,000 per year. The mean (SD) SF-12 PCS and MCS scores were 52.1 (8.3) and 49.0 (9.1), respectively, close to population norms.

**Table 1:** Demographics and Health Status

**Age (years), mean (SD)**	46.0 (12.9)
	
**Sex**	
Male, N (%)	83 (38)
Female, N (%)	135 (62)
	
**Ethnicity**	
Caucasian, N (%)	143 (66)
African Americans, N (%)	62 (28)
Other, N (%)	13(6)
	
**Marital Status**	
Married, N (%)	96 (44)
Separated, N (%)	10 (5)
Divorced, N (%)	48 (22)
Widowed, N (%)	11 (5)
Single, N (%)	52 (24)
	
**Education**	
Did not finish high school, N (%)	6 (2)
High school graduate, N (%)	43 (20)
Started but did not complete college, N (%)	70 (32)
College graduate, N (%)	59 (27)
Post graduate, N (%)	38 (18)
	
**Annual Income**	
< $12,000, N (%)	34 (16)
$12,000-25,000, N (%)	29 (13)
$25,000-50,000, N (%)	67 (31)
$50,000-75,000, N (%)	46 (21)
> $75,000, N (%)	31 (14)
Preferred not to say	11 (5)
	
**Health Status**	
SF-12 Physical Component Summary, mean (SD)	52.1 (8.3)
SF-12 Mental Component Summary, mean (SD)	49.0 (9.1)
Health Assessment Questionnaire-Disability Index, mean (SD)	0.12 (0.30)

### Health Utilities for PsA Health States

Health ratings and utilities for PsA health states were generally inversely related to the severity of the PsA health state. Mean (SD) RS scores ranged from 63.7 (20.3) for mild PsA to 20.2 (14.4) for severe PsA (Table 2). Mean TTO scores ranged from 0.78 (0.24) for mild PsA to 0.29 (0.31) for severe PsA, indicating a willingness to trade up to, on average, 22% (= [1-0.78] × 100%) of life expectancy with mild PsA to 71% (= [1-0.29] × 100%) of life expectancy with severe PsA in exchange for perfect health. Mean SG scores ranged from 0.82 (0.24) for mild PsA to 0.48 (0.34) for severe PsA. Thus, participants were willing to accept an average risk of death as high as 18% (= [1-0.82] × 100%) with mild PsA to 52% (= [1-0.48] × 100%) with severe PsA for a chance at perfect health.

**Table 2:** Psoriatic Arthritis Utilities

Health State	Number of respondents	Mean	SD
**Mild PsA**			
RS	70	63.7	20.3
TTO	70	0.78	0.24
SG	70	0.82	0.24
**Moderate PsA**			
RS	78	45.7	16.9
TTO	78	0.58	0.31
SG	78	0.67	0.30
**Severe PsA**			
RS	66	20.2	14.4
TTO	66	0.29	0.31
SG	66	0.48	0.34

SD: standard deviation; PsA: psoriatic arthritis; RS: rating scale (range: 0-100); TTO: time tradeoff (range: 0.0-1.0); SG: standard gamble (range: 0.0-1.0).

### Health Utilities for SSc Health States

Health ratings and utilities for SSc health states were also inversely related to the severity of the health state. Mean (SD) RS scores ranged from 69.7 (15.3) for mild SSc to 25.3 (15.2) for severe SSc with lung disease (Table 3). Mean (SD) TTO scores ranged from 0.80 (0.25) for mild SSc to 0.36 (0.31) for severe SSc with lung disease, indicating a willingness to forgo a mean of 20% (with mild SSc) to 64% (with severe SSc and lung disease) of life expectancy in exchange for perfect health. Mean SG scores ranged from 0.81 (0.26) for mild SSc to 0.50 (0.31) for severe SSc and 0.51 (0.32) for severe SSc with lung disease. Thus, participants were willing to accept an average risk of death as high as 19% (mild SSc) to 50% (severe SSc with lung involvement) and 51% (without lung involvement) for a chance at perfect health.

**Table 3:** Scleroderma Utilities

Health State	Number of respondents	Mean	SD
**Mild SSc**			
RS	94	69.7	15.3
TTO	94	0.80	0.25
SG	94	0.81	0.26
**Moderate SSc**			
RS	172	54.9	15.8
TTO	172	0.68	0.28
SG	172	0.76	0.27
**Moderate SSc with lung involvement**			
RS	177	45.3	15.5
TTO	177	0.59	0.3
SG	177	0.68	0.28
**Severe SSc**			
RS	120	30	16.3
TTO	120	0.37	0.29
SG	120	0.50	0.31
**Severe SSc with lung involvement**			
RS	90	25.3	15.2
TTO	90	0.36	0.31
SG	90	0.51	0.32

SD: standard deviation; PsA: psoriatic arthritis; RS: rating scale (range: 0-100); TTO: time tradeoff (range: 0.0-1.0); SG: standard gamble (range: 0.0-1.0).

### Comprehension and Empathy

When asked about their understanding of the computer-assisted utility exercises, 129 (59%) of the subjects rated it as very clear, 70 (32%) as clear, 15 (7%) as neither clear nor confusing, 3 (2%) as confusing, and 1 (1%) as very confusing. Of the 210 (out of 218) participants, 140 (67%) and 65 (31%) were able to empathize very much or a little bit, respectively, with a person with the PsA or SSc health states, and only 5 (2%) could not imagine themselves as a person with PsA or SSc.

## Discussion

PsA and SSc are chronic, often disabling diseases with a detrimental impact on HRQoL [27,28]. Assessing health values (utilities) - ideally from the general public - is an essential element for economic evaluations of healthcare interventions in these and other diseases.

There are two different approaches to obtaining utilities from the general public. First, patients with a particular disease can fill out a health state classification instrument that uses population-assigned weights to calculate utility scores for particular health states. A variety of measures are available for this purpose, including the EQ-5 D, the QWB-SA, the Health Utilities Index, and the SF-6 D [16,29-32]. The SF-6 D, which is derived from the SF-36 Health Survey, is a health state classification instrument that uses population weights assessed in the U.K. Using data from two different studies, we analyzed SF-6 D scores in patients with either limited or diffuse SSc of varying severity [8]. The mean (SD) SF-6 D scores in the two studies were 0.61 (0.12) and 0.64 (0.13) on a scale ranging from 0.29 to 1.00. Neither study assessed the severity of patients' SSc.

The second method is to ask people from the general public directly to value health states common to a particular disease. The advantage of this method over the health state classification measurement method is that specific aspects of the disease can be described in various ways (e.g., with pictures or videos) beyond simple brief written descriptions available in a generic health status measure [33,34]. To obtain community utilities for PsA and SSc, we interviewed 218 participants in a mid-size city in the U.S. The proportion of Caucasians (66%) in our sample is representative of the 2005 U.S. census and the proportion of African-Americans (28%) is representative of the city in which the study took place. The health status of our participants, as captured by the SF-12, was similar to that of the U.S. general population [16,35].

The utility approach explicitly acknowledges that preferences are used to express the relative importance of various health outcomes [21]. Understanding the concepts of the SG and TTO may be difficult for some subjects. To assess that, we asked our participants about their understanding of the health value assessment exercise; 91% rated it as clear or very clear. In addition, 98% of participants were able to empathize with the persons described in the PsA and SSc health states. Both of these findings lend confidence to our results. Furthermore, the health utility scores for mild, moderate and severe PsA and SSc support the construct validity of the utility measures in that more severe health states were assigned lower utilities than were less severe health states. In addition, as described in the literature previously, TTO and SG scores were generally relatively higher than RS scores, as the RS does not involve tradeoffs against an external metric such as time or risk of death. Our findings are consistent with previously published data that suggest that utility values derived using the SG are higher than those using the TTO for more severe health states, whereas the reverse may be true for less severe health states [36-38].

Several of our findings warrant particular attention. First, subjects assigned similar disutility to mild SSc and PsA health states, but moderate and severe PsA was assigned a greater disutility (lower utility) than moderate and severe SSc with or without lung involvement. This finding may be due to the public's perception that having thickened skin (from SSc) is more acceptable than having erythematous, pruritic scaly skin lesions (from PsA). Alternatively, it is possible that participants did not fully understand the full spectrum of differences between the two diseases, especially as related to mortality. This also may be reflective of the relatively young population of respondents, or their relatively low education and/or socioeconomic status.

Our data corroborate previous research showing that the general public assigns greater disutility to hypothetical health states in most, but not all, circumstances than do patients experiencing those health states [39]. When health utilities were assessed in 107 patients with SSc of varying severity, the mean RS, TTO, and SG scores were 64.3, 0.76, and 0.74, respectively [2], scores that fell in the least severe SSc categories in our study. Health utilities have also been assessed in patients with psoriasis but without arthritis [14]. In those patients, the RS, TTO, and SG correlated inversely with extent of skin involvement. Specifically, the median RS score was 0.76 for patients having less than 10% of their skin surface involved vs. 0.34 for those having more than 30% skin involvement. Corresponding median TTO and SG values were 0.99 for both measures (< 10% of skin involved) vs. 0.75 for both measures (> 30% of skin involved). Although patient-derived utilities are valuable for decision making involving individual patients, for cost-effectiveness analyses, the U.S. Public Health Service Panel on Cost-Effectiveness in Health and Medicine has recommended using utilities assessed from the general public [15]. Although the general public tends to underestimate utilities of patients with a given condition, the Panel reasoned that community utilities for hypothetical health states represent the public's interest better [40,41]. Also, members of the general public are potential future patients [42].

We had hypothesized that the general public would assign a lower utility to diffuse SSc, which manifests as greater skin thickening compared with limited SSc, for otherwise similar health states. Surprisingly, this was not the case. In other words, to the general public, the extent of skin thickening does not significantly affect the value of SSc health states. This finding may due to the way the health states were described or to limited power to detect differences in utilities for limited vs. diffuse SSc; alternatively, when assigning utilities, subjects may have focused more on ability to perform avocational and day-to-day activities rather than the extent of skin involvement. Health state classification systems by necessity are limited in the number of attributes they cover. Because we based our health state descriptions on the QWB-SA, we did not include additional clinical manifestations of PsA and SSc. It is possible that had we described additional aspects of severe SSc (e.g., finger contractures, painful ulcers, and painful calcinosis), then the utility for severe SSc might have been lower, perhaps even lower than the utility for PsA. Capturing those manifestations may have elucidated differences in utilities related to extraarticular features of PsA and to extent of involvement of SSc, but also may have generated too many health states for the subjects to be able to process. We also did not include prognostic information in describing the health states for diffuse vs. limited disease and in describing the associated lung disease. Prognostic information in the form of life expectancy is already captured in calculating QALYs; thus, the convention is to exclude prognostic information from the health state description *per se *so as to avoid double counting [43]. Still, although diffuse SSc is more severe than limited SSc, in a previous study of patients with SSc we found that SG scores were actually higher among patients with the diffuse subtype (mean score 0.79 vs. 0.69 for patients with limited SSc) and that TTO scores were similar (0.76 for diffuse SSc and 0.77 for limited SSc) in the two groups [2].

Our study had several limitations. Participants were not selected randomly - rather, they were a convenience sample of respondents to newspaper ads and posters in one city. Thus, it is unlikely that the sample is truly representative of the U.S. population, especially given our low proportion of Hispanic patients. Nationally, Hispanics represent 14% of the U.S. population [44]. Although health utilities generally don't differ by ethnicity, further research is necessary [45]. Second, we sought to recruit subjects who had not experienced symptoms of inflammatory arthritis. By excluding patients with SSc, PsA, and other inflammatory arthritides from the utility assessment exercise, the results may be slightly non-representative of the general population. We believe that any such bias is minimal, given that with a sample size of 218, one would only expect to have 3-4 patients with these conditions [46,47].

## Conclusion

These limitations notwithstanding, this study provides community-based quality-of-life weights for PsA and SSc health states. Understanding and taking into account these values is important for determining treatment strategies. As such, these findings could have implications regarding comparative effectiveness research.

## Competing interests

The authors declare that they have no competing interests.

## Authors' contributions

DK: PI of the study, study design, supervision and manuscript preparation. TF, PK, RMK, MHE, RDH: manuscript preparation. SG: data collection (study coordinator) and manuscript preparation. ACL: statistical analysis and manuscript preparation. JT: study design, supervision and manuscript preparation. All authors read and approved the final manuscript.

## Appendix Health State Descriptions

### Mild Psoriatic Arthritis

Imagine that you:

• Travel in a car or by public transportation without difficulty.

• Do not have problems walking around.

• Independently perform all major activities (e.g., working, moderate exercise or household chores).

• Independently perform all self-care activities (e.g., eating, dressing and bathing).

However, sometimes your hands and feet are painful, swollen and stiff when you do these things.

Also, you have a health condition that has caused:

• Raised, reddish skin covered by silvery-white scale on the elbows, knees, lower back, and scalp.

• Your skin to itch often.

• Your skin to sometimes crack and bleed.

### Moderate Psoriatic Arthritis

Imagine that you:

• Travel in a car or by public transportation with some difficulty.

• Have problems walking around and sometimes need to use a cane.

• Can *not *independently perform all major activities (e.g., working, moderate exercise or household chores).

• Can independently perform all self-care activities (e.g., eating, dressing and bathing).

However, often your hands and feet are painful, swollen and stiff.

Also, you have a health condition that has caused:

• Raised, reddish skin covered by silvery-white scale on the elbows, knees, lower back, and scalp.

• Your skin to itch often.

• Your skin to often crack and bleed.

### Severe Psoriatic Arthritis

Imagine that you:

• Travel in a car or by public transportation with much difficulty.

• Have problems walking around and sometimes need to use a cane.

• Can *not *Independently perform all major activities (e.g., working, moderate exercise or household chores).

• Can *not *independently perform all self-care activities (e.g., eating, dressing and bathing).

However, your hands and feet are constantly very painful, swollen and stiff.

Also, you have a health condition that has caused:

• Raised, reddish skin covered by silvery-white scale on the elbows, knees, lower back, and scalp.

• Your skin to itch often.

• Your skin to often crack and bleed.

• Discomfort that may *keep you up at night*.

### Mild Limited Scleroderma

Imagine that you:

• Travel in a car or by public transportation without difficulty.

• Do *not *have problems walking around.

• Independently perform all major activities (e.g., working, moderate exercise or household chores).

• Independently perform all self-care activities (e.g., eating, dressing and bathing).

However, sometimes your JOINTS are painful, swollen and stiff.

Also, you have a health condition that has:

• Caused thickening and hardening of your skin.

• Left you with slight scarring on your face, hands, arms, and legs.

### Moderate Limited Scleroderma

Imagine that you:

• Travel in a car or by public transportation with some difficulty.

• Have problems walking around and sometimes need to use a cane.

• Can *not *independently perform all major activities (e.g., working, moderate exercise or household chores).

• Can independently perform all self-care activities (e.g., eating, dressing and bathing).

However, often your JOINTS are painful, swollen and stiff.

Also, you have a health condition that has:

• Caused thickening and hardening of your skin.

• Left you with some scarring on your face, hands, arms, and legs.

### Moderate Limited Scleroderma with Lung Disease

Imagine that you:

• Travel in a car or by public transportation with some difficulty.

• Have shortness of breath which causes some problems in walking around (you need to stop and catch your breath sometimes).

• Can *not *Independently perform all major activities (e.g., working, moderate exercise or household chores).

• *Can *Independently perform all self-care activities (e.g., eating, dressing and bathing).

However, often your JOINTS are painful, swollen and stiff.

Also, you have a health condition that has:

• Caused thickening and hardening of your skin.

• Left you with some scarring on your face, hands, arms, and legs.

### Severe Limited Scleroderma

Imagine that you:

• Travel in a car or by public transportation with much difficulty.

• Have problems walking around and sometimes need to use a cane.

• Can *not *Independently perform all major activities (e.g., working, moderate exercise or household chores).

• Can *not *independently perform all self-care activities (e.g., eating, dressing and bathing).

However, your JOINTS are constantly very painful, swollen and stiff.

Also, you have a health condition that has:

• Caused thickening and hardening of your skin.

• Left you with some scarring on your face, hands, arms, and legs.

### Severe Limited Scleroderma with Lung Disease

Imagine that you:

• Travel in a car or by public transportation with much difficulty.

• Have shortness of Breath which causes some problems in walking around (you need to *stop and catch *your breath and *use a cane *sometimes).

• Can *not *Independently perform all major activities (e.g., working, moderate exercise or household chores).

• Can *not *independently perform all self-care activities (e.g., eating, dressing and bathing).

Your JOINTS are constantly very painful, swollen and stiff.

Also, you have a health condition that has:

• Caused thickening and hardening of your skin.

• Left you with some scarring on your face, hands, arms, and legs.

Diffuse scleroderma health state descriptions were the same as above except for the statement: "Health condition has left you with significant scarring on large portions of your face, hands, arms, and legs."
